# Alphavirus nsP3 as a multifunctional orchestrator of virus-host interplay

**DOI:** 10.1128/jvi.01779-25

**Published:** 2026-06-15

**Authors:** Ana Alice Pimenta-Pereira, Bailey Lubinski, Rafael Kroon Campos

**Affiliations:** 1 Department of Microbiology and Immunology, College of Veterinary Medicine, Cornell University 5922, Ithaca, New York, USA; Indiana University Bloomington, Bloomington, Indiana, USA

**Keywords:** alphavirus, nsP3, host factors, virus-host interaction

## Abstract

Alphaviruses can cause polyarthralgia and encephalitis and pose an escalating global health threat to humans and animals. They are transmitted to vertebrates mainly by infected mosquitoes, leading to host alternation that drives an evolutionary arms race between the virus and host, during which the virus is selected to efficiently utilize the cellular factors of both hosts for replication. Alphaviruses encode the information necessary for replication in their genomic viral RNA, which is translated into nonstructural proteins, such as nonstructural protein 3 (nsP3). nsP3 is a remarkably multifunctional protein that promotes replication of viral RNAs and translation through diverse mechanisms. Among these mechanisms, nsP3 serves as a hub to recruit host proteins, acts as an RNA-binding protein, removes post-translational modifications from host and viral proteins, and modulates cellular pathways and host immune responses. nsP3 oligomerizes into helical scaffolds present in both viral replication complexes and nsP3-positive condensates that form during infection. Although the importance of nsP3 is evident, many of its roles remain less well understood compared to those of other alphavirus proteins. Here, we provide an overview of the current knowledge on how nsP3 promotes virus replication and pathogenesis and influences host range specificity. Further studies on nsP3 are needed to elucidate its mechanisms of action in the alphavirus replication cycle, which may ultimately support new strategies for outbreak surveillance, prediction, and infection countermeasures.

## INTRODUCTION

Alphaviruses are enveloped, positive-sense RNA viruses in the *Togaviridae* family and *Alphavirus* genus (1, 2). They are transmitted mainly by mosquitoes, which become infected while feeding on a viremic vertebrate host and later transmit the virus during subsequent blood meals (3). For a successful transmission cycle, the virus must replicate in both the mosquito and the vertebrate host. Alphaviruses infect a broad range of vertebrates, including humans, other mammals, birds, fish, and reptiles (4, 5). Their emergence, defined as the first appearance or spread into new regions, and reemergence, a resurgence after a period of decline (6), pose an increasing threat to public and animal health, as well as a substantial economic burden (7, 8). Alphaviruses are classified by the type of disease caused. Encephalitic alphaviruses include Venezuelan, western, and eastern equine encephalitis viruses (VEEV, WEEV, and EEEV). Arthritogenic alphaviruses include Chikungunya virus (CHIKV), o'nyong-nyong virus (ONNV), and Ross River virus (RRV) (1), among others.

**Fig 1:**
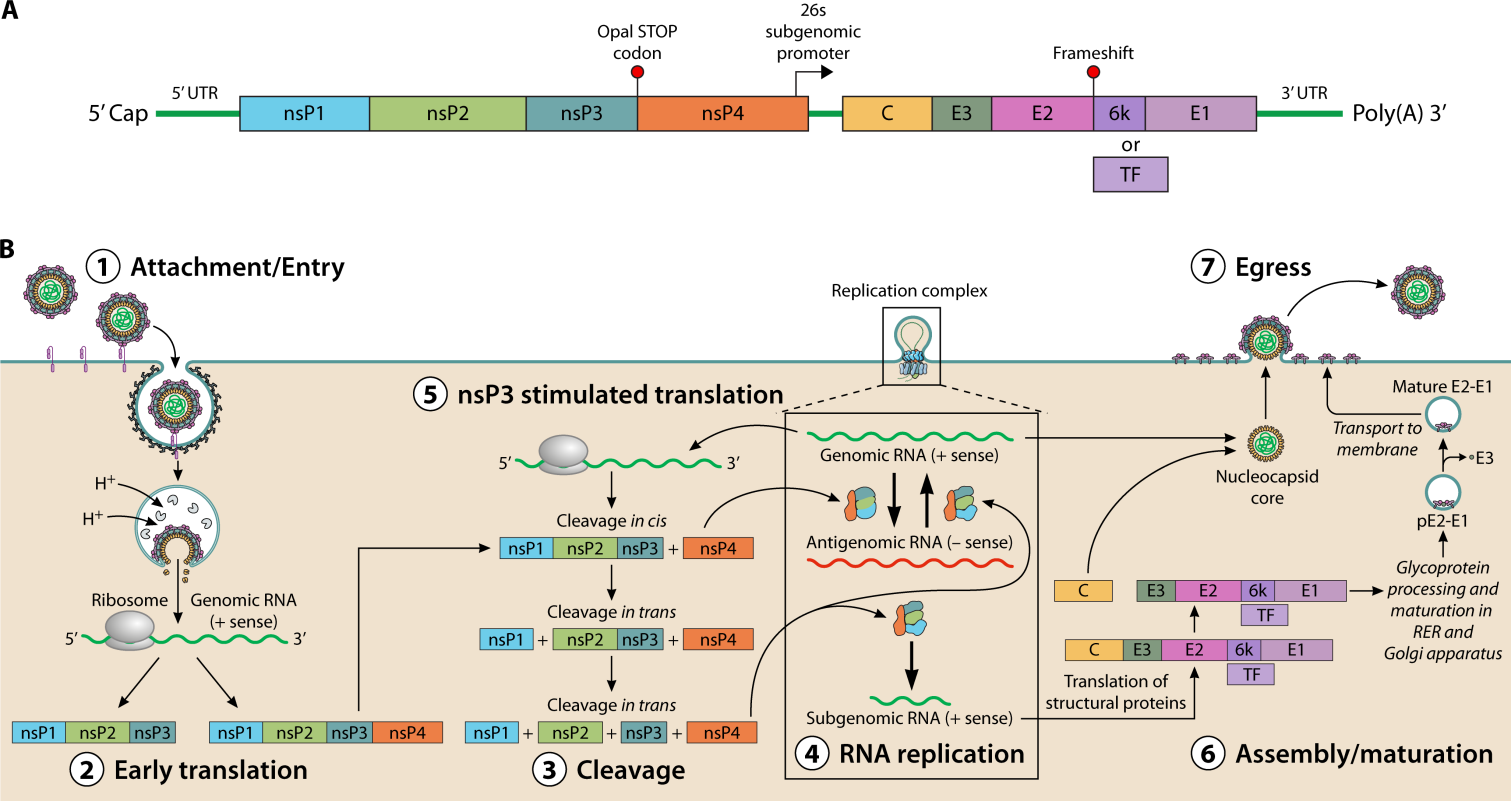
Alphavirus genome organization and the roles of nsP3 in the intracellular replication cycle. (**A**) Representation of the virus genome showing coding and non-coding regions, the opal stop codon feature, sgRNA promoter, and location of the ribosome frameshift, which can result in production of the transframe (TF) protein. (**B**) Virus attachment and receptor binding to susceptible cells trigger entry via clathrin-mediated endocytosis (step 1). Acidification of the endosomes leads to low-pH-mediated fusion and genome release into the cytosol, with cathepsins potentially playing a role in facilitating entry. Nonstructural proteins are translated from the genomic RNA (gRNA) independently of nsP3 (step 2). Alphavirus nonstructural polyprotein and its cleavage products (step 3), along with host proteins recruited by nsP3, induce formation of the replication complex spherules, where the agRNA, sgRNA, and additional gRNA are produced (step 4). nsP123 + 4 has negative-sense replicase activity, whereas the cleaved products are important for positive-sense replicase. After nsP3 accumulates through translation and replication rounds, it stimulates translation by recruiting host factors (step 5). Structural polyprotein is then translated from the sgRNA, being subsequently processed into capsid (by its autoprotease function), and envelope proteins, which are translocated to the endoplasmic reticulum, processed by cellular signalases, glycosylated, and transported to the Golgi complex, where furin cleavage removes E3 from E2. Capsid binds gRNA to form the nucleocapsid, which incorporates the glycoproteins, and virus egress occurs by budding from the plasma membrane. Credit: ScEYEnce Studios.

Following the entry of alphaviruses into the host cell, the translation of the alphavirus genome yields the nonstructural proteins (nsPs) nsP1, nsP2, nsP3, and nsP4 (Fig. 1A). This precedes the production of the subgenomic RNA (sgRNA), which is replicated from the antigenomic RNA (agRNA), translated, and processed to produce the structural proteins: capsid and three envelope proteins, along with the 6k peptide and the transframe protein (2, 9, 10). To replicate successfully within host cells, alphaviruses rely on the nsPs (Fig. 1B) via a myriad of mechanisms, including the formation of the replication complex (11, 12), viral RNA (vRNA) capping (13, 14), RNA helicase activity (15, 16), proteolytic separation of the nsPs (17, 18), and RNA-dependent RNA polymerase function (19). While all nsPs are essential for virus replication, nsP3 stands out for its multiple critical yet poorly characterized functions (20, 21).

Current knowledge of nsP3 supports its influence on viral fitness (21, 22), pathogenesis (23), and host-range specificity (24–26). Intracellularly, nsP3 (27–29) promotes translation (30, 31) and is essential for vRNA replication (27–29), which are key stages of the intracellular replication cycle. It has also been hypothesized to support virus nucleocapsid assembly (21, 32, 33), although a rigorously designed experiment is required to test this possibility. Although not strictly required for replication in all models, nsP3 also performs immune evasion functions in both mosquito and vertebrate hosts (26, 34, 35). Here, we review the features of nsP3, emphasizing the need for a better mechanistic understanding of its multifunctionality and interactions with vertebrate and mosquito host factors.

## nsP3 STRUCTURE, FUNCTIONS, AND INTRACELLULAR LOCALIZATION

**Fig 2:**
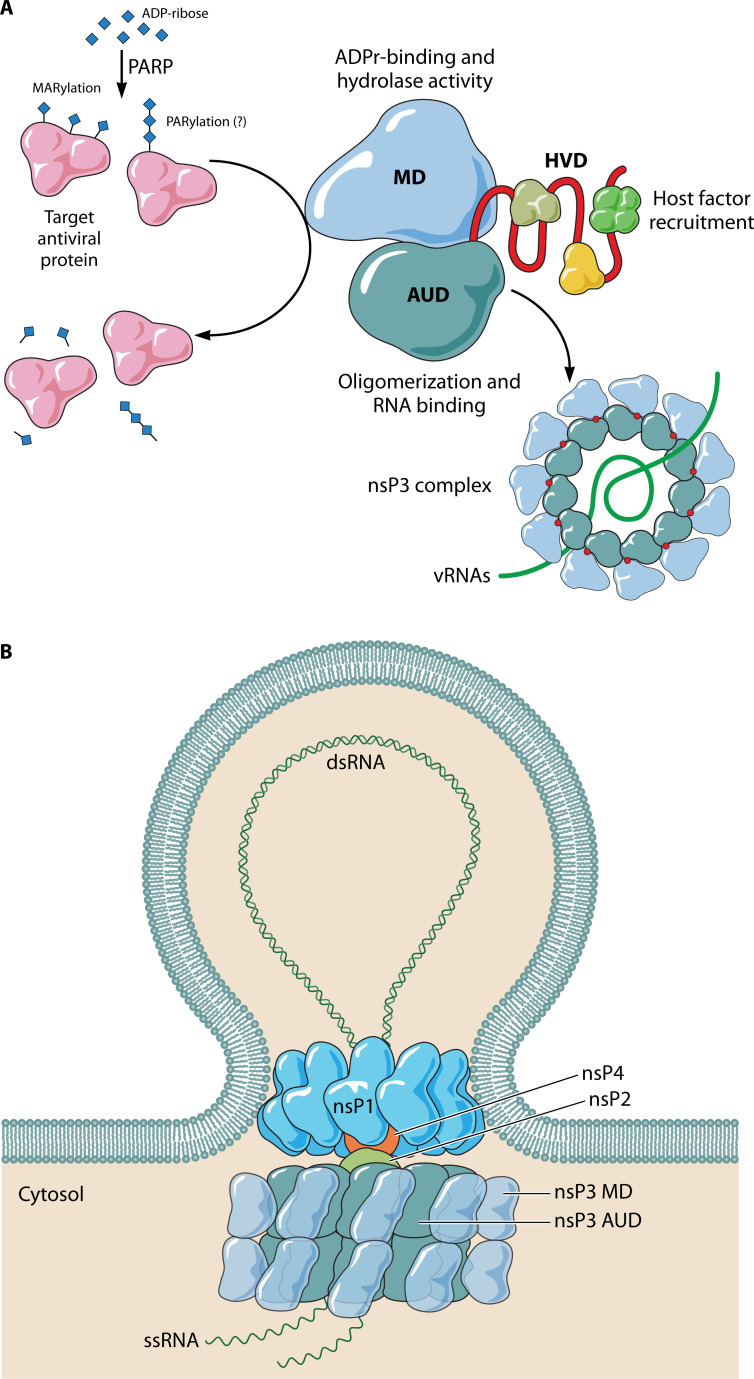
Main functions of the nsP3 domains. (**A**) nsP3 is composed of three domains: MD, AUD, and HVD. MD has ADPr-binding and hydrolase activities that together work to remove ADPr marks from host and viral proteins, which are added by host proteins such as PARPs and can participate in antiviral signaling. Both of these activities have been shown to be important directly for RNA replication through an unknown mechanism. Removal of ADP ribosylation can also counteract the immune pathways dependent on this modification. The AUD functions in binding vRNAs and in self-oligomerization, which is important for nsP3’s functions in vRNA translation and replication. The HVD specializes in binding and recruiting host factors to support vRNA translation and replication, as well as modulating granules and disrupting antiviral cellular functions. (**B**) nsP3 is an essential part of the replication complex in the form of an oligomeric crown on the cytosolic face of the replication complex. The HVD has not been resolved in this structure, but it is likely present in flexible conformations that allow it to recruit various host factors needed for RNA replication. Credit: ScEYEnce Studios.

The alphavirus nsP3 contains three domains: the N-terminal macrodomain (MD), the alphavirus-unique domain (AUD), and the C-terminal hypervariable domain (HVD) (Fig. 2A) (25, 36). The MD is a highly conserved domain found in several virus families, including *Coronaviridae*, *Hepeviridae*, and *Togaviridae* (37–39). It consists of a central, twisted six-stranded beta sheet surrounded by several alpha helices and contains a pocket that binds adenosine diphosphate-ribose (ADPr) (Fig. 2A) (29). The second domain, the AUD, is unique to and highly conserved among alphaviruses. It contains a zinc-binding motif in which four conserved cysteine residues coordinate a zinc ion, a feature that forms an RNA-interaction surface essential for RNA binding (21, 36). The AUD promotes nsP3 oligomerization into tubular structures. The HVD is the least conserved nsP3 domain in alphaviruses and is intrinsically disordered and flexible (40). Its flexible structure (27) allows it to sample diverse conformations and interact with a range of host factors (Fig. 2A) (30).

As an essential component for viral RNA replication, nsP3 oligomerizes to form a two-start tubular structure with helical symmetry, similar to a crown, on the cytosolic side of the replication complex (Fig. 2B). A slight shift of the helix rise results in the formation of superposed nsP3 rings, with 11.37 protomers per turn. This nsP3 structure connects to a membrane-anchored dodecameric nsP1 crown in a misaligned manner, preventing rigid interlocking and allowing movement (32, 41, 42). The nsP4 viral polymerase, in complex with the viral nsP2 helicase-protease, is located in the central pore of the nsP1 crown (43). The nsP1 crown has a plug-like density and is responsible for vRNA capping and for gating the replication complex, posited to control access to this viral organelle, and ensuring the exit of properly capped viral RNA (44). Conversely, the nsP3 crown has a mostly empty cavity, proposed to interact with genomic RNA (gRNA), sgRNA, and agRNA (32).

The domains of nsP3 have both independent and synergistic functions to facilitate its diverse roles. The MD belongs to the MacroD subclass and directly binds mono-ADP-ribosylated proteins, glutamate and aspartate residues, and removes this modification (Fig. 2A), which is added mainly by cellular poly(ADPr) polymerases (PARPs), thereby altering their activity (35). Mutagenesis studies of CHIKV MD in neural cells found that both the ADPr binding and hydrolase activities are essential for vRNA replicase activity (29). ADPr binding itself was found to facilitate nonstructural protein synthesis and establishment of replication complexes, whereas hydrolase activity is important for double-stranded RNA (dsRNA) amplification in replication complexes (29, 35, 45). The requirement for ADPr binding suggests that CHIKV co-opts host proteins to initiate vRNA translation and replication (29). However, the mechanisms and specific host protein targets for each of these functions remain mostly elusive. Many studies also show that ADP-ribosylated proteins can have antiviral effects, so MD’s activity is also important for immune system evasion (37, 46–50).

The AUD directly interacts with the subgenomic promoter region, 5′UTR, and 3′UTR of the vRNA through its zinc-binding motif, and mutagenesis of any of the conserved cysteine residues completely disrupts vRNA replication (21, 32). Mutations of residues around the cysteines result in cell-specific phenotypes, and one double mutation in CHIKV P247A/V248A shows a notably specific effect on synthesis of the sgRNA, but not gRNA (21), suggesting that different amino acids in AUD may control the specificity of binding to gRNA versus sgRNA. AUD-promoted nsP3 oligomers are likely required for these RNA-binding functions and are essential for successful infection. However, the ratio of monomeric to oligomeric nsP3 remains unknown, as does whether the monomer has functions of its own.

The HVD has roles in binding a myriad of host factors contributing to vRNA translation and replication, and similar to the other two domains, it is essential for infection. Unlike the other two domains, the HVD can accommodate larger insertions and deletions with minimal impact on replication, which may facilitate adaptation to selective pressures imposed by replication in both vertebrate and mosquito hosts (51). However, disrupting certain motifs where important host factors bind can lead to non-replicative phenotypes (52–55). The structure and roles of nsP3 throughout infection highlight its versatility of functions at different stages of the virus replication cycle.

### Subcellular localization, stress granules (SGs), and virus-modified granules (VMGs)

**Fig 3:**
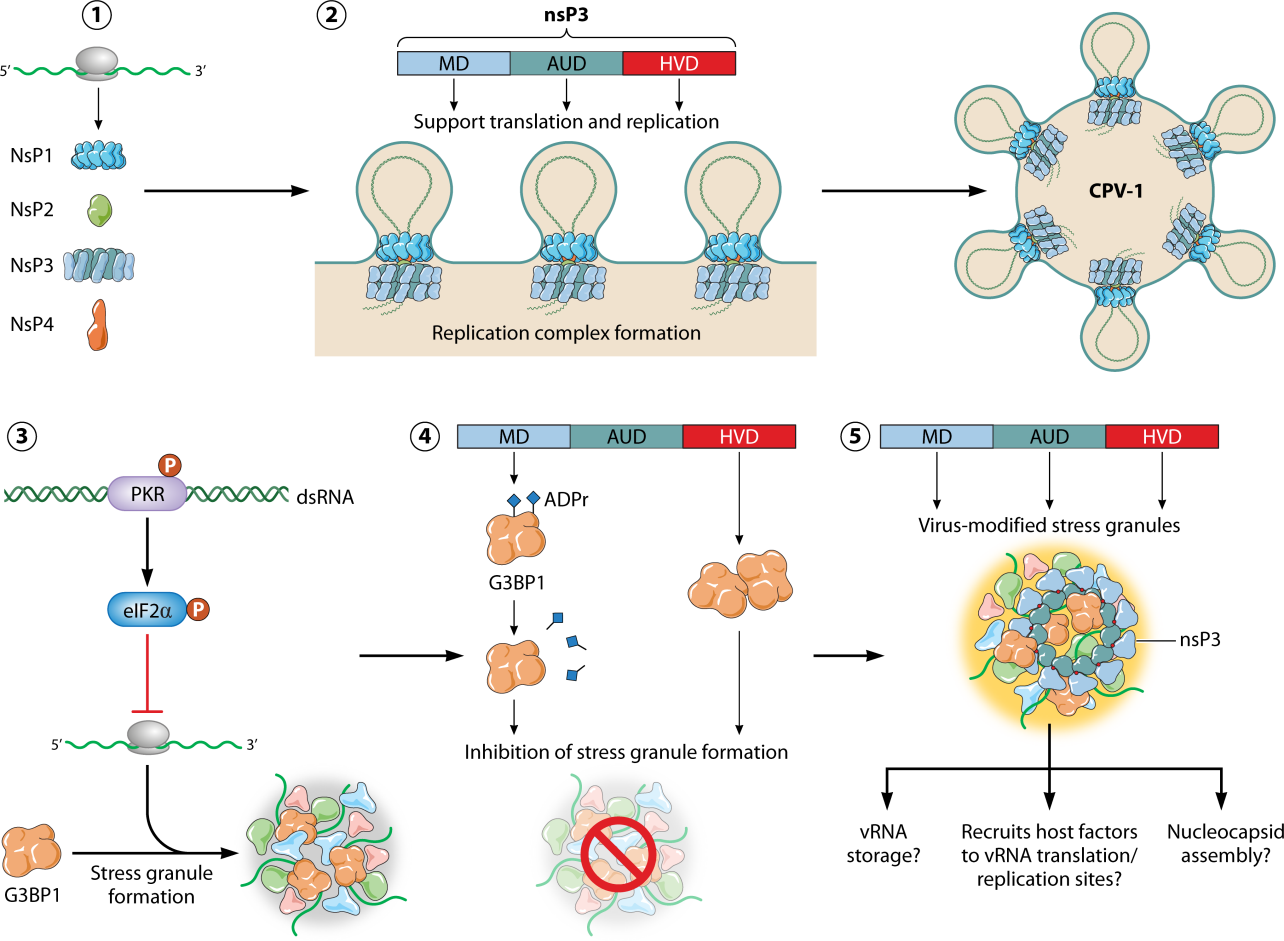
nsP3 subcellular localization and temporal modulation of cellular programs. Early in infection, nonstructural proteins are translated (1) and are critical for the formation of the replication complex spherules where vRNA replication takes place. nsP3 is an integral part of the replication complex and recruits host factors that are essential for vRNA replication and promote more efficient translation (2). nsP3 promotes the transition of replication complexes from the plasma membrane to CPV-1 by binding p85 and activating the AKT pathway (2). The replication of vRNA leads to dsRNA, which is recognized by PKR, triggering the phosphorylation of eIF2α and inhibiting canonical cap-dependent protein synthesis. This leads to the formation of canonical SGs containing the host protein G3BP1 (3). Alphaviruses use different mechanisms to inhibit SGs, some of which are dependent on nsP3. The MD removal of ADP ribosylation from G3BP1 and the HVD recruitment of G3BP1 have been shown to have the potential to contribute to the inhibition of SGs (4) and also appear to be critical for the transition from SGs to VMGs (5). Similar to SGs, VMGs contain G3BP1, but unlike SGs, they also contain nsP3 and capsid. They are hypothesized to function in vRNA safe storage during stress responses, in the recruitment of host factors to vRNA translation and replication sites, and potentially play a role in promoting efficient virus assembly. Credit: ScEYEnce Studios.

nsP3 accumulates in different locations in the cytoplasm of the cell. One of the main sites of nsP3 accumulation is in the replication complexes, which are present at the plasma membrane early in infection (Fig. 3), and as the infection progresses, transitions in an nsP3-dependent manner to structures known as cytopathic vacuoles type I (CPV-I) (56–59). Another prominent site of nsP3 accumulation is the VMGs (Fig. 3), which have many similarities to SGs (34, 45, 60).

SGs are membrane-free cellular structures that form in response to various stressors, including virus infection. SG formation typically depends on stress-induced phosphorylation of eukaryotic initiation factor 2α (eIF2α), a key negative regulator of global protein synthesis from cellular messenger RNA (mRNA) (61). In alphavirus infections, dsRNA formed during replication is thought to be recognized by protein kinase R (PKR) (Fig. 3), leading to eIF2α phosphorylation and reduction in translation of many cellular mRNAs (62). Hallmark components of SG are stalled mRNA, translation initiation factors, small ribosomal (40S) subunits, and RNA-binding proteins (RBPs) (63). Although the function of SG in infection remains largely unknown, several SG proteins can either promote or inhibit alphavirus replication by regulating RNA molecules’ stability, localization, and translation (64).

While alphaviruses initially trigger SG assembly, they subsequently suppress SG formation, rendering cells resistant even to chemical SG inducers such as sodium arsenite (62, 64). Several non-mutually exclusive mechanisms have been proposed for how alphaviruses block SG formation. These mechanisms include HVD sequestration of Ras-GTPase-activating protein SH3-domain-binding proteins (G3BPs) (64, 65), MD removal of ADP-ribosylation of G3BP1 (45), and nsP3 regulation of eIF2α localization and phosphorylation (61). There are also mechanisms independent of nsP3, such as alphavirus-induced transcriptional and translational shutoffs (62), and capsid competing with G3BP1 for binding to RNA (66). There appears to be a temporal component, correlated with the virus life cycle (Fig. 1B), in which transcriptional and translational shutoffs will happen first (62), followed by accumulation of nsP3 (45), and then capsid accumulation (66), which could represent redundant or complementary mechanisms for SG inhibition.

After the initial SG formation is blocked, VMG containing nsP3 forms (Fig. 3) (45), whose composition and function are poorly understood. Strikingly, nsP3 tubular structures are much longer inside VMG compared to replication complexes (32). The MD promotes VMG formation by de-ADP-ribosylating G3BP1 (45), while the HVD is essential for recruiting nsP3 to these complexes (67). VMG share similarities with canonical SG, as they associate with RBPs such as G3BP1 (45, 68), Y-box binding protein 1 (YBX1), and heat shock 70 kDa (HSC70) (68), but they also contain viral proteins nsP3 and capsid, as well as vRNA (62, 64). In CHIKV infection, these VMG were reported to lack translation factors, which are typical components of classic SG (34, 45). However, a study using Semliki Forest virus (SFV) showed active viral translation at VMG sites (69), which could suggest differences in the strategies used by these viruses, or reflect the temporally dynamic nature of these compartments. Although these condensate modifications are postulated to play important roles in infection, it has been difficult to separate their functions from other functions played by viral nsP3 and host G3BP1.

## HUMAN HOST FACTORS THAT INTERACT WITH nsP3

The structural features and domains discussed in the previous section enable nsP3 to interact with and co-opt a diverse range of human host proteins to support virus replication. Some of these host proteins share specific domains or motifs that mediate nsP3 binding (20, 28, 70). nsP3 interactors can promote or restrict virus replication, whereas others have no measurable impact, underscoring the importance of validating which interactions are biologically relevant. Many of these interactions remain to be mechanistically characterized, and direct binding has been demonstrated for only a few host proteins. We grouped nsP3-interacting proteins into validated direct interactors with mapped binding motifs (Table 1) and proteins with indirect, unresolved (validated binding but unknown whether it is direct or indirect), or putative interactors (Table 2).

**TABLE 1:** Alphavirus nsP3 validated direct interactors^*a*^

Cellular function		Protein	Virus	Putative function during infection	Best described motif	Reference(s)
RNA-binding	Stress granules	G3BP family: G3BP1, GRBP2	CHIKV, SINV, SFV, VEEV, EEEV, WEEV, RRV, GETV, MAYV	Proviral	NTF2L domains bind to FG(D/S)(I/L/F) in the HVD Two FDGF motifs in CHIKV, EILV, ONNV FGSF and FGDI in SINV FGAP in MAYV FGDI and FGDK in GETV LITF in EEEV	(5, 30, 31, 34, 52, 58, 60, 64, 65, 68, 71–77)
		FXR family: FMR1, FXR1, FXR2	CHIKV, SINV, SFV, VEEV, EEEV	Proviral	Agenet-like domain binds to the carboxy terminus (residues 170 and 227) in EEEV HVD PPGVNRVITREEFEA minimal motif in VEEV HVD	(22, 31, 52, 78, 79)
		YBX1	CHIKV, SINV, SFV, VEEV, ONNV	Proviral	CSD and CTD domains interact with SSELLT motif in CHIKV HVD	(68, 80)
Chaperones	Histone chaperones	NAP family: NAP1L1 and NAP1L4	CHIKV	Proviral		
					Two motifs in CHIKV HVD, one upstream and one downstream of the G3BP-binding motifs	(28, 78, 81–83)
					1st: TTMELSHPPISFGAP	
					2nd: DDLTDSDWSTCPDTDD	
	
					Binding is phosphorylation-dependent, requiring the phosphorylated HVD; CK2 mediates the phosphorylation.	
		NPM1/B23	CHIKV	Antiviral	ADP-ribosylation-linked interface binds residues N24, Y114 in CHIKV MD	(50)
Cytoskeleton and membrane remodeling	Scaffold proteins	CD2AP, SH3KBP1	CHIKV, SFV, VEEV, EEEV	Proviral	Two motifs in CHIKV HVD, with related motifs in SFV and VEEV	
					1st: P(I/V)(P/A)PPRGRNLTVT	(22, 27, 28, 79, 84)
					2nd: PMASVR	
		FHL1	CHIKV, ONNV	Proviral	FHL1 LIM1 domain binds to TVTCDEREGNITPMASVRFFRAELCPVVQETAETRDTAMSLQAPP in CHIKV HVD	(28, 70, 85–87)
	Endocytic adaptor proteins	BIN 1	CHIKV, SINV, SFV	Proviral	BIN1 SH3 domain bind to PXXPXRpXR in SINV, and SFV HVD M1 (PVAPPR) and M2 (PMASVR) in CHIKV HVD	(27, 28, 31, 53, 85, 88)
Kinases	Catalytic serine/threonine kinase	IKKβ	VEEV; WEEV	Proviral	Phosphorylates VEEV nsP3, at residues 204/205, 142, and 134/135	(89–93)
	Signaling adaptors	p85 regulatory subunit of PI 3-kinase	CHIKV, SFV, RRV	Proviral	SH2 domain of p85 binds YXXM motif in SFV and RRV HVD	(89–93)

^*a*^ Direct interactors based on curated alphavirus literature and UniProt cellular function for each host protein.

**TABLE 2:** Alphavirus nsP3 indirect, unresolved, or putative interactors^*a*^

Cellular function	Functional subgroup	Protein	Virus	Study type	Reference(s)
RNA-binding	Stress granules/ RNA granules	IGF2BP1	EEEV; SINV	HVD pulldown/proteomics; CRISPR KO/KI functional validation; virus replication and imaging assays | HVD immunoprecipitation/proteomics; mutagenesis;	(52, 78)
		IGF2BP2	EEEV; SINV	HVD immunoprecipitation/proteomics; mutagenesis; knockout/functional validation	(78)
		IGF2BP3	EEEV; VEEV; CHIKV; SINV	HVD immunoprecipitation/proteomics; mutagenesis; knockout/functional validation	(78)
		TRBP	SINV	Subcellular fractionation; affinity purification; MALDI-TOF proteomics	(68)
		USP10	SFV	miniTurbo proximity labeling; proteomics; siRNA knockdown	(31)
		YBX3	CHIKV; SINV	HVD immunoprecipitation/proteomics; mutagenesis; knockout/functional validation	(78)
		CIRBP	CHIKV	Co-immunoprecipitation-LC-MS/MS in infected Huh7 cells	(82)
		UBAP2L	SFV	miniTurbo proximity labeling; proteomics	(31)
		ATXN2L	SFV	miniTurbo proximity labeling; proteomics	(31)
		CAPRIN1	SFV	miniTurbo proximity labeling; proteomics	(31)
		NUFIP2	SFV	miniTurbo proximity labeling; proteomics	(31)
	Nucleolar/ ribosome biogenesis factors	B23.2	SINV	GFP-tagged nsP3 complex isolation; co-immunoprecipitation/mass spectrometry	(73)
		MYBBP1A	CHIKV; SINV	HVD immunoprecipitation/proteomics; mutagenesis; knockout/functional validation	(78)
		NUCLEOLIN	SINV; CHIKV	GFP-tagged nsP3 affinity purification-mass spectrometry (AP-MS); localization/imaging | Co-immunoprecipitation-LC-MS/MS in infected Huh7 cells	(58, 82)
		FBL	CHIKV	Co-immunoprecipitation-LC-MS/MS in infected Huh7 cells	(82)
	Post-transcription regulators	INTS6	SINV	GFP-tagged nsP3 AP-MS; localization/imaging	(58)
		PCBP1/hnRNP E1	SFV	Magnetic fractionation of replication organelles; SILAC quantitative proteomics; functional silencing	(94)
		PSF	SINV	GFP-tagged nsP3 AP-MS; localization/imaging	(58)
		XAB2	SFV	miniTurbo proximity labeling; proteomics; siRNA knockdown	(31)
		hnRNP A0	SINV	GFP-tagged nsP3 complex isolation; co-immunoprecipitation/mass spectrometry	(73)
		hnRNP A1	SINV	GFP-tagged nsP3 AP-MS; localization/imaging | GFP-tagged nsP3 complex isolation; co-immunoprecipitation/mass spectrometry	(58, 73)
		hnRNP A1-like	VEEV	HA-tagged nsP3 immunoprecipitation-LC-MS/MS; siRNA knockdown	(95)
		hnRNP A2/B1	SINV	GFP-tagged nsP3 AP-MS; localization/imaging | GFP-tagged nsP3 complex isolation; co-immunoprecipitation/mass spectrometry	(58, 73)
		hnRNP A3	SINV	GFP-tagged nsP3 AP-MS; localization/imaging | GFP-tagged nsP3 complex isolation; co-immunoprecipitation/mass spectrometry	(58, 73)
		hnRNP C	CHIKV, SFV	Co-immunoprecipitation-LC-MS/MS in infected Huh7 cells | Magnetic fractionation of replication organelles; SILAC quantitative proteomics; functional silencing	(82, 94)
		hnRNP G	SINV	GFP-tagged nsP3 AP-MS; localization/imaging	(58)
		hnRNP K	SFV; SINV	Magnetic fractionation of replication organelles; SILAC quantitative proteomics; functional silencing | Comparative 2D electrophoresis; co-immunoprecipitation; colocalization/RNA co-IP	(94, 96)
		hnRNP M	SFV	Magnetic fractionation of replication organelles; SILAC quantitative proteomics; functional silencing	(94)
		hnRNP U	SINV	GFP-tagged nsP3 complex isolation; co-immunoprecipitation/mass spectrometry	(73)
		NUDT21	CHIKV	Co-immunoprecipitation-LC-MS/MS in infected Huh7 cells	(82)
		NONO	CHIKV	Co-immunoprecipitation-LC-MS/MS in infected Huh7 cells	(82)
		RBM22	SFV	miniTurbo proximity labeling; proteomics; siRNA knockdown	(31)
		RC3H1	SFV	miniTurbo proximity labeling; proteomics	(31)
		STAU1	SFV	miniTurbo proximity labeling; proteomics	(31)
		YTHDF2	SFV	miniTurbo proximity labeling; proteomics	(31)
		YTHDF3	SFV	miniTurbo proximity labeling; proteomics	(31)
		XRN1	SFV	miniTurbo proximity labeling; proteomics	(31)
	Proteins associated with the translation machinery	eIF2S1 (eIF2α)	CHIKV	Co-immunoprecipitation-LC-MS/MS in infected Huh7 cells; co-IP/immunofluorescence validation	(82)
		eIF4G1	SFV	miniTurbo proximity labeling; proteomics; siRNA knockdown	(31)
		RACK1/GNB2L1	SINV	GFP-tagged nsP3 AP-MS; localization/imaging	(58)
		eEF1A	SINV	GFP-tagged nsP3 complex isolation; co-immunoprecipitation/mass spectrometry	(73)
		eEF2	SINV	miniTurbo proximity labeling; proteomics; siRNA knockdown | GFP-tagged nsP3 AP-MS; localization/imaging	(31, 58)
		eIF5A	CHIKV	Co-immunoprecipitation-LC-MS/MS in infected Huh7 cells	(82)
		eIF2S3	CHIKV	Co-immunoprecipitation-LC-MS/MS in infected Huh7 cells	(82)
		eEF1B2	CHIKV	Co-immunoprecipitation-LC-MS/MS in infected Huh7 cells	(82)
		eEF1A2	CHIKV	Co-immunoprecipitation-LC-MS/MS in infected Huh7 cells	(82)
		eIF2A	SFV	miniTurbo proximity labeling; proteomics	(31)
		eIF4B	SFV	miniTurbo proximity labeling; proteomics	(31)
		eIF4G2	SFV	miniTurbo proximity labeling; proteomics	(31)
		eIF4G3	SFV	miniTurbo proximity labeling; proteomics	(31)
		eIF5	SFV	miniTurbo proximity labeling; proteomics	(31)
		eRF3A	SFV	miniTurbo proximity labeling; proteomics	(31)
		eEF1G	CHIKV	Co-immunoprecipitation-LC-MS/MS in infected Huh7 cells	(82)
		PRRC2C	SFV	miniTurbo proximity labeling; proteomics	(31)
		SERBP1	SFV	miniTurbo proximity labeling; proteomics	(31)
	RNA helicases	DDX1	VEEV	HA-tagged nsP3 immunoprecipitation-LC-MS/MS; siRNA knockdown	(95)
		DDX17	SINV	GFP-tagged nsP3 AP-MS; localization/imaging | HVD immunoprecipitation/proteomics; mutagenesis; knockout/functional validation	(58, 78)
		DDX3	VEEV	HA-tagged nsP3 immunoprecipitation-LC-MS/MS; siRNA knockdown	(95)
		DDX5	SINV, CHIKV	GFP-tagged nsP3 AP-MS; localization/imaging | HVD immunoprecipitation/proteomics; mutagenesis; knockout/functional validation | Co-immunoprecipitation-LC-MS/MS in infected Huh7 cells	(58, 78)
		DDX6	SINV	HVD immunoprecipitation/proteomics; mutagenesis; knockout/functional validation	(78)
		DHX9	SINV	GFP-tagged nsP3 AP-MS; localization/imaging	(58)
	Ribosomal proteins	RPL10	SINV	GFP-tagged nsP3 complex isolation; co-immunoprecipitation/mass spectrometry	(73)
		RPL10A	SINV; VEEV	GFP-tagged nsP3 complex isolation; co-immunoprecipitation/mass spectrometry Subcellular fractionation; affinity purification; MALDI-TOF proteomics | HA-tagged nsP3 immunoprecipitation-LC-MS/MS; siRNA knockdown	(73, 95)
		RPL15	SINV	GFP-tagged nsP3 AP-MS; localization/imaging	(58)
		RPL18A	SINV	GFP-tagged nsP3 AP-MS; localization/imaging	(58)
		RPL23A	SINV	Subcellular fractionation; affinity purification; MALDI-TOF proteomics	(68)
		RPL3	SINV	GFP-tagged nsP3 AP-MS; localization/imaging	(58)
		RPL4	SINV	Subcellular fractionation; affinity purification; MALDI-TOF proteomics	(68)
		RPL5	SINV	Subcellular fractionation; affinity purification; MALDI-TOF proteomics	(68)
		RPL6	SINV; VEEV	Subcellular fractionation; affinity purification; MALDI-TOF proteomics | HA-tagged nsP3 immunoprecipitation-LC-MS/MS; siRNA knockdown	(68, 95)
		RPL7	SINV	GFP-tagged nsP3 complex isolation; co-immunoprecipitation/mass spectrometry | Subcellular fractionation; affinity purification; MALDI-TOF proteomics	(68, 73)
		RP L7A	SINV	Subcellular fractionation; affinity purification; MALDI-TOF proteomics	(68)
		RPL8	SINV	Subcellular fractionation; affinity purification; MALDI-TOF proteomics	(68, 95)
		RPLP0	VEEV	HA-tagged nsP3 immunoprecipitation-LC-MS/MS; siRNA knockdown	(95)
		RPS10	SINV	GFP-tagged nsP3 AP-MS; localization/imaging	(58)
		RPS18	SINV	GFP-tagged nsP3 complex isolation; co-immunoprecipitation/mass spectrometry	(73)
		RPS3	SINV	GFP-tagged nsP3 AP-MS; localization/imaging	(58)
		RPS8	SINV; VEEV	GFP-tagged nsP3 AP-MS; localization/imaging | HA-tagged nsP3 immunoprecipitation-LC-MS/MS; siRNA knockdown	(58, 95)
		RPS9	SINV	GFP-tagged nsP3 AP-MS; localization/imaging | GFP-tagged nsP3 complex isolation; co-immunoprecipitation/mass spectrometry	(58, 73)
Chaperones	Heat shock proteins	DNAJC9	CHIKV; SINV	HVD immunoprecipitation/proteomics; mutagenesis; knockout/functional validation	(78)
		GRP78	SINV	GFP-tagged nsP3 AP-MS; localization/imaging | Subcellular fractionation; affinity purification; MALDI-TOF proteomics	(58, 68)
		HSC70	SINV	GFP-tagged nsP3 AP-MS; localization/imaging | GFP-tagged nsP3 complex isolation; co-immunoprecipitation/mass spectrometry | Subcellular fractionation; affinity purification; MALDI-TOF proteomics	(58, 68)
		HSP90	CHIKV	Pull-down; mass spectrometry; microscopy; siRNA/inhibitor validation	(97)
		HSPA1B	EEEV; VEEV; CHIKV; SINV	HVD immunoprecipitation/proteomics; mutagenesis; knockout/functional validation	(78)
		SERPINH1	CHIKV	Co-immunoprecipitation-LC-MS/MS in infected Huh7 cells	(82)
Cytoskeleton and membrane remodeling	Cytoskeletal structural/ motor proteins	ATCB	SINV	GFP-tagged nsP3 complex isolation; co-immunoprecipitation/mass spectrometry	(73)
		TUBB	SINV	GFP-tagged nsP3 complex isolation; co-immunoprecipitation/mass spectrometry	(73)
		CAPZA1	VEEV; EEEV	Comparative HVD pull-down/co-isolation proteomics; knockout/functional validation | HVD immunoprecipitation/proteomics; mutagenesis; knockout/functional validation	(52, 78)
		CAPZA2	EEEV; VEEV	HVD immunoprecipitation/proteomics; mutagenesis; knockout/functional validation	(78)
		CAPZB	VEEV; EEEV	Comparative HVD pull-down/co-isolation proteomics; knockout/functional validation | HVD immunoprecipitation/proteomics; mutagenesis; knockout/functional validation	(52, 78)
		DES	SINV	GFP-tagged nsP3 complex isolation; co-immunoprecipitation/mass spectrometry	(73)
		MAP1B	VEEV	HA-tagged nsP3 immunoprecipitation-LC-MS/MS; siRNA knockdown	(95)
		MYH9	SINV	GFP-tagged nsP3 complex isolation; co-immunoprecipitation/mass spectrometry	(73)
		MYLIP	SINV	GFP-tagged nsP3 complex isolation; co-immunoprecipitation/mass spectrometry	(73)
		PRPH	SINV	GFP-tagged nsP3 AP-MS; localization/imaging	(58)
		VIM	SINV	GFP-tagged nsP3 complex isolation; co-immunoprecipitation/mass spectrometry	(73)
	Endocytic adaptor proteins	CLTC	EEEV	HVD immunoprecipitation/proteomics; mutagenesis; knockout/functional validation	(78)
		SH3GL1	SFV	miniTurbo proximity labeling; proteomics; siRNA knockdown	(31)
		SNX33	EEEV	HVD immunoprecipitation/proteomics; mutagenesis; knockout/functional validation	(78)
		SNX9	EEEV	HVD immunoprecipitation/proteomics; mutagenesis; knockout/functional validation	(78)
		SNAPIN	CHIKV	Bioinformatics/Computational analysis; GST pull-down	(98)
	Scaffold proteins	14-3-3 z	SINV	Subcellular fractionation; affinity purification; MALDI-TOF proteomics	(73)
		14-3-3 β/α	SINV	GFP-tagged nsP3 complex isolation; co-immunoprecipitation/mass spectrometry	(73)
		14-3-3 γ	SINV	GFP-tagged nsP3 complex isolation; co-immunoprecipitation/mass spectrometry	(58, 73)
		14-3-3 ε	SINV	GFP-tagged nsP3 AP-MS; localization/imaging | GFP-tagged nsP3 complex isolation; co-immunoprecipitation/mass spectrometry | Subcellular fractionation; affinity purification; MALDI-TOF proteomics	(58, 68, 73)
		14-3-3 ζ	SINV	GFP-tagged nsP3 AP-MS; localization/imaging	(58)
		14-3-3 ζ/δ	SINV	GFP-tagged nsP3 complex isolation; co-immunoprecipitation/mass spectrometry	(73)
		14-3-3 η	SINV	GFP-tagged nsP3 AP-MS; localization/imaging	(58)
		14-3-3 τ	SINV	GFP-tagged nsP3 complex isolation; co-immunoprecipitation/mass spectrometry	(73)
		AHNAK	SFV	miniTurbo proximity labeling; proteomics; siRNA knockdown	(31)
		CKAP4	SINV	GFP-tagged nsP3 AP-MS; localization/imaging	(58)
		PLEC	SINV; VEEV	GFP-tagged nsP3 complex isolation; co-immunoprecipitation/mass spectrometry | HA-tagged nsP3 immunoprecipitation-LC-MS/MS; siRNA knockdown	(73, 95)
		RDX	CHIKV	HVD immunoprecipitation/proteomics; mutagenesis; knockout/functional validation	(78)
		SASH1	SFV; SINV; CHIKV	SH3-domain interaction screen; binding validation/mutagenesis	(53)
Immune-related proteins	Innate/ stress signaling	ANKRD17	SFV	miniTurbo proximity labeling; proteomics; siRNA knockdown	(31)
		IKKβ	VEEV; WEEV	Functional inhibition/knockout study; proteomics	(89, 90)
		JUN	CHIKV	HVD immunoprecipitation/proteomics; mutagenesis; knockout/functional validation	(78)
		PGAM5	EEEV; VEEV; CHIKV	HVD immunoprecipitation/proteomics; mutagenesis; knockout/functional validation	(78)
		S100A4	EEEV; VEEV	HVD immunoprecipitation/proteomics; mutagenesis; knockout/functional validation	(78)
		SK2	CHIKV	Colocalization/RNA association; AP-MS of SK2 interactome; siRNA/inhibitor studies	(99)
Metabolic/ mitochondrial proteins	Metabolic and mitochondrial proteins	ASS1	CHIKV	Co-immunoprecipitation-LC-MS/MS in infected Huh7 cells; co-IP/immunofluorescence validation	(82)
		GSTM1	SINV	GFP-tagged nsP3 AP-MS; localization/imaging	(58)
		LYPLA1/2	SINV	Subcellular fractionation; affinity purification; MALDI-TOF proteomics	(68)
		PKM	CHIKV	Co-immunoprecipitation-LC-MS/MS in infected Huh7 cells; co-IP/immunofluorescence validation	(82)
		SLC25A13	EEEV; VEEV	HVD immunoprecipitation/proteomics; mutagenesis; knockout/functional validation	(78)
		SLC25A5	EEEV; VEEV; SINV	HVD immunoprecipitation/proteomics; mutagenesis; knockout/functional validation | GFP-tagged nsP3 AP-MS; localization/imaging	(58, 78)
		PAICS	CHIKV	Co-immunoprecipitation-LC-MS/MS in infected Huh7 cells	(82)
		ARG1	CHIKV	Co-immunoprecipitation-LC-MS/MS in infected Huh7 cells	(82)
		OAT	CHIKV	Co-immunoprecipitation-LC-MS/MS in infected Huh7 cells	(82)
Nuclear/ chromatin regulators	DNA and chromatin regulators	DNMT1	SINV	GFP-tagged nsP3 AP-MS; localization/imaging	(58)
		HELQ	SINV	GFP-tagged nsP3 AP-MS; localization/imaging	(58)
		HIST1H1C	CHIKV	HVD immunoprecipitation/proteomics; mutagenesis; knockout/functional validation	(78)
		N4BP2L2	CHIKV	Bioinformatics/Computational analysis; GST pull-down	(98)
Signaling	Signaling regulators	SH3BP-5	SINV	GFP-tagged nsP3 AP-MS; localization/imaging	(58)
		WDR48	SINV	Comparative HVD pull-down/co-isolation proteomics; knockout/functional validation | HVD immunoprecipitation/proteomics; mutagenesis; knockout/functional validation	(52, 78)
		CAMK2D	CHIKV	Co-immunoprecipitation-LC-MS/MS in infected Huh7 cells	(82)
	Ubiquitin ligase	NEDD4	CHIKV	Targeted co-IP; targeted pull-down; siRNA/overexpression functional validation; nsP3 domain mapping/truncation	(100)
ADP-ribosylated proteins	ADP-ribose MAR/PARylated proteins	PARP-1	SINV	Co-immunoprecipitation; domain mapping; inhibitor/replication assays	(48)
		PARP-7	SFV;SINV	HA-IP-tagged nsP3; overexpression functional assay; siRNA/inhibition studies; MD nsP3 mutant	(46)
		PARP-12	SFV;SINV	HA-IP-tagged nsP3; overexpression functional assay; siRNA/inhibition studies; MD nsP3 mutant	(46)
		PARP-15	SFV;SINV	HA-IP-tagged nsP3; overexpression functional assay; siRNA/inhibition studies; MD nsP3 mutant	(46)

^*a*^ Indirect, unresolved or putative interactors based on curated alphavirus literature and UniProt cellular function for each host protein.

### Alphavirus nsP3 direct interactors

Host protein interactions are not evenly distributed across nsP3. The vast majority of these interactors bind the HVD, and this clustering likely reflects this domain’s role in recruiting host factors, but could also be a bias from more extensive experimental characterization of this domain (Table 1).

#### RNA-binding proteins

RBPs are commonly co-opted by nsP3 to promote vRNA replication (30, 52, 78, 80) and translation (30, 65). The most well-studied are G3BP1 and G3BP2 (G3BP1/2) (34, 58, 60, 65, 68), whose cellular functions include acting as central switches for assembling SGs (101, 102), regulating the stability and translation of mRNAs (103), and playing central roles in immune signaling such as RIG-I (104, 105) and cGAS (106, 107) pathways. Due to their role in recruiting key antiviral factors to SG and contributing to antiviral responses, these proteins exhibit antiviral activity against many viral families (108). In stark contrast, most alphaviruses not only counteract this antiviral defense (28, 65, 109), but most of the arthritogenic alphaviruses have evolved to co-opt these proteins as proviral factors (28, 65).

G3BP1/2 share the same domains, including the NTF2-like domain (NTF2L) and an arginine-/glycine-rich (RGG) domain (108). The NTF2L of G3BP1 directly interacts with the HVD at FGDF motifs and other similar, non-canonical motifs (Table 1). These include two FGDF motifs in CHIKV (60, 71), FGSF and FGDI motifs in Sindbis virus (SINV) (5, 52), and the FGDF and FGAP motifs in MAYV (109). The RGG RNA-binding domain promotes SFV and CHIKV translation by recruiting 40S ribosomal subunits (30). Accordingly, G3BP1/2 have been linked in SFV to replication-complex formation and recruitment of translation-initiation machinery (30, 72). Beyond promoting vRNA translation, G3BP1/2 may facilitate ribosome release from incoming gRNA to enable negative-strand synthesis and support nsP3-dependent pre-replication complex assembly by recruiting viral gRNA to replication sites (52, 65). In CHIKV-infected cells, nsP3-G3BP1/2 foci appear first at the plasma membrane without detectable viral RNA, then associate with single-stranded viral RNA, and later with dsRNA (52), consistent with replication complex maturation.

G3BP1 and G3BP2 can have redundant functions. For CHIKV and MAYV, individual or combined depletion of G3BP1 and G3BP2 reduces vRNA accumulation and negative-strand RNA synthesis, supporting a proviral role for both proteins. Similarly, CRISPR-based studies in SINV indicate that G3BP1/2 act as partially redundant proviral factors (52). However, their individual contributions may require further investigation. One study reported that G3BP1 knockdown induced increased compensatory expression of G3BP2, but not the reverse (102). Another study showed that combined G3BP1/2 depletion produced the greatest replication defect, although G3BP2 single depletion had a stronger effect than G3BP1 single depletion for both SINV and CHIKV (65).

While many arthritogenic alphaviruses recruit G3BP1/2 via FGDF motifs in nsP3, the nsP3 of encephalitic alphaviruses such as VEEV, EEEV, and WEEV contain C-terminal Agenet-like domain-binding motifs that directly recruit fragile X-related (FXR) proteins, including FXR1, FXR2, and fragile X messenger ribonucleoprotein 1 (FMR1), for replication and into VMDs (5, 52, 78). Arthritogenic alphaviruses recruit FMR1 indirectly via G3BP1/2 (5), suggesting partial functional redundancy between these two protein families. EEEV illustrates this dual strategy, directly binding and co-opting proteins of the G3BP and FXR families (78). For this virus, mutation of the FXR-binding motif reduces neurovirulence in mice, while disruption of both FXR and G3BP1/2 binding motifs completely abolishes replication (22). Similarly, VEEV recruits FXR proteins via a 33-aa C-terminal repeat motif, and FXR depletion slows replication and decreases dsRNA-positive replication complexes (52). Mutation of the FXR-binding motif attenuates disease in neonatal mice but has only limited effects on viremia and neuroinvasion in adult mice (79). Overall, FXR recruitment supports early replication complex formation and contributes to alphavirus pathogenesis, but the degree of its impact varies between viral species and disease models. Defining these differences will require rescue experiments and tissue-specific mechanistic studies.

Another critical RBP that interacts with nsP3 is YBX1. It was initially identified as a putative nsP3 interactor in SINV (68) and has since been validated as a direct interactor of CHIKV nsP3 (80). Loss of YBX1 has been shown to reduce replication of several alphaviruses, including CHIKV, SINV, SFV, VEEV, and ONNV (80), indicating a proviral role. Although YBX1 can interact directly with nsP3 via CSD and CTD motifs, its recruitment during infection depends on RNA and its RNA-binding activity, suggesting a complex interaction mechanism. YBX1 may bind both nsP3 and viral RNA (80). However, it remains unclear whether its role depends on simultaneous or sequential association with both nsP3 and RNA or whether one precedes and enables the other.

#### Chaperones

The nucleosome assembly protein (NAP) 1-like family of chaperones is also proposed to have important roles during early stages of alphavirus infection. In CHIKV, NAP1L1 and NAP1L4 are recruited through phosphorylated motifs in the nsP3 HVD. The motif phosphorylation is mediated by CK2α/CK2 (28, 81). Disruption of the NAP1L-binding region impairs RNA infectivity and replication, particularly in vertebrate cells, whereas restoration of this sequence rescues replication competence (28).

In contrast, another chaperone, nucleophosmin 1 (NPM1), appears to antagonize CHIKV replication through a distinct mechanism centered on the MD activity (50, 110). During infection, NPM1 relocalizes from the nucleus to the cytoplasm, where its ADP-ribosylated form engages the nsP3 MD, particularly at residues N24 and Y114 (50). Nuclear-retained NPM1 mutants lose antiviral activity, indicating that cytoplasmic localization is necessary for viral restriction, likely because viral replication occurs in the cytosol (50). Depletion of NPM1 promotes increased viral protein accumulation, whereas overexpression suppresses nsP3 and E2 levels, dsRNA foci, and infectious virus yields (50). The mechanistic basis of this restriction remains unresolved, particularly with respect to NPM1 engagement of nsP3, the role of NPM1 ADP-ribosylation in binding or antiviral activity, and which step of the replication cycle is being inhibited. NPM1 may restrict infection by promoting the expression of antiviral interferon-stimulated genes (50).

#### Cytoskeleton and membrane remodeling proteins

Cellular architecture undergoes substantial remodeling during alphavirus infection, and nsP3 is thought to support these rearrangements by recruiting SH3 domain-containing proteins, such as CD2-associated protein (CD2AP), SH3 domain-containing kinase-binding protein 1 (SH3KBP1) (27, 84), close homologs, and the bridging Integrator 1 (BIN1) (88). These proteins interact with overlapping proline-rich motifs in HVD. BIN1 was initially mapped to the upstream PIPPPR SH3-binding motif in SFV, SINV, and CHIKV (53), whereas CD2AP and SH3KBP1 were first linked to the downstream PMASVR-containing motif in CHIKV (84). Comprehensive mutagenesis mapping of CHIKV nsP3 showed that two SH3-binding sites, M1 (PVAPPR) and M2 (PMASVR), together recruit BIN1, CD2AP, and SH3KBP1 as a shared adaptor module; mutation of both sites abolishes binding to the three factors and strongly impairs replication initiation, showing a predominantly redundant proviral role (27). This recruitment appears to be further modulated by HVD phosphorylation, since substitution of Ser/Thr residues with alanine in either the N-terminal (320–396) or C-terminal (411–519) region of the CHIKV nsP3 HVD weakens, but does not abolish, binding (85).

While the molecular requirements for recruitment are becoming clearer, the mechanisms through which CD2AP and SH3KBP1 facilitate replication remain insufficiently characterized. Given their roles in actin-associated and endocytic pathways (111, 112), these proteins may link nsP3-containing complexes to host cytoskeletal and membrane-trafficking machinery at early replication sites (27). In CHIKV, disruption of the SH3-binding domain prevents the recruitment of CD2AP and SH3KBP1 to replication-associated structures and reduces the infectivity of transfected RNA (84).

BIN1 functions in early endocytic events and binds CHIKV and SFV nsP3 with higher affinity than its canonical cellular ligand, dynamin (88). This high-affinity interaction is mediated not only by the core motif, but also by an extended arginine-rich region that engages the broad acidic surface of the BIN1 SH3 domain (88). Functionally, BIN1 is important for SFV and SINV replication, as disruption of nsP3 SH3 binding impairs BIN1 recruitment, delays CPV formation, reduces RNA replication, and attenuates infection (53).

Interactions with cytoskeletal proteins also influence viral tropism and pathogenesis. The four and a half LIM domains protein 1 (FHL1) is a direct nsP3 interactor for CHIKV (70, 86). It binds HVD residues 424–456, a region that partially overlaps with the CD2AP-interacting motif (70), and it is linked to infection in joints and skeletal muscle, two important tissues for the arthritogenic disease phenotype (86). The reduction in negative-strand RNA synthesis, together with the failure to form replicative spherules in FHL1-deficient cells, strongly suggests that FHL1 has a proviral role, supporting the early phase of the CHIKV replication cycle (86). Consistent with this role, loss of FHL1 reduces infection by CHIKV and ONNV, but not by other alphaviruses such as RRV and MAYV or by flaviviruses (86), suggesting that FHL1 interaction may be virus-specific.

Loss of FHL1 markedly restricts infection in otherwise CHIKV-susceptible cells, while ectopic expression of FHL1 restores susceptibility in resistant cells (86). CHIKV can still replicate in FHL1-deficient cells, albeit less efficiently (70), indicating that the presence of FHL1 may be an important enhancer of infection rather than an absolute requirement. Experiments with neonatal FHL1-deficient mice showed reduced viral loads in joints and muscles with minimal muscle pathology (86). In adult immunocompetent mice, FHL1 has been shown to promote musculoskeletal disease, and disruption of the nsP3-FHL1 interaction yielded an avirulent CHIKV mutant (87). Taken together, these results support the role of FHL1 in pathogenesis.

#### Kinases

nsP3 HVD is a main protein target of phosphorylation in alphaviruses (113, 114). IKKβ is a kinase with the most well-characterized mechanism for adding this modification. During VEEV infection, NF-κB signaling is activated, and IKKβ shifts into infection-specific, lower-molecular-weight complexes (89, 90). IKKβ directly phosphorylates VEEV nsP3 at residues 204/205, 142, and 134/135, promoting negative-strand RNA synthesis and viral replication (89). Phosphomimetic analyses show that phosphorylation at 204/205 is critical for both negative-strand synthesis and replication, whereas phosphorylation at 134/135 supports replication without affecting negative-strand RNA production (89). A phosphomimetic mutation at residue 142 does not rescue replication, suggesting that phosphorylation at this site may be necessary but not sufficient (89). IKKβ inhibition results in reduced viral titers and vRNA levels, whereas IKKβ overexpression increases virus production, and IKKβ inhibitors improve survival in VEEV-infected mice (90).

Beyond direct nsP3 phosphorylation, alphavirus HVD motifs directly engage host signaling kinase pathways. In SFV, replication complexes form at the plasma membrane and are later internalized into CPV-I compartments (115). This trafficking phenotype was linked to an nsP3-mediated activation of the PI3K-AKT-mTOR pathway (56, 91). In SFV and RRV, strong activation of this pathway is driven by a YXXM motif in the nsP3 HVD, which recruits the p85 regulatory subunit of PI3K and promotes AKT activation (92). By contrast, CHIKV lacks this motif and induces weaker AKT signaling, consistent with its slower movement of replication complexes from the plasma membrane to CPV-I compartments (91). SFV infection promotes increased glycolysis, pentose phosphate pathway activity, TCA cycle flux, and fatty acid synthesis, indicating that AKT signaling supports biosynthetic pathways required for efficient virion production (92, 116). Inhibition of this signaling reduces these phosphorylation events and impairs viral replication mainly at late stages, suggesting that PI3K-AKT activation is important later in infection (91, 92). In RRV, loss of AKT hyperactivation has little effect on replication *in vitro* but reduces disease severity *in vivo*, supporting a role in pathogenesis (92). In contrast, SINV lacks a YXXM motif and shows low and transient PI3K-AKT activation (92, 117). However, glycolysis and pentose phosphate pathway activity still contribute to efficient replication of SINV (92, 116), suggesting metabolic remodeling can occur independently of AKT activation. Different papers reported distinct replication outcomes upon mTORC1 inhibition, with some showing decreased SINV replication (117) and others showing increased CHIKV replication due to increased translation (118).

#### Alphavirus nsP3 indirect, unresolved, or putative interactors

Earlier work on alphavirus nsP3 relied on targeted approaches such as co-immunoprecipitation (73, 78, 95), affinity capture (52, 58, 96, 97), and fraction-based studies (53, 89, 90, 96, 99, 100, 119). Advances in protein-protein interaction mapping through mass spectrometry have extended the list of putative vertebrate nsP3 interactors (Table 2) (31, 82, 120).

Similar to validated interactors, most unresolved interactors are proposed to bind the HVD (25, 28, 31, 52, 84). However, thousands of host proteins are known to be modified by ADPr marks (121) and could be targets of MD, which must interact with the substrate protein for ADPr removal, and are potential partners of MD (37). Despite this potential, a major knowledge gap remains regarding the specific host factors that interact with the MD, and from our list, only NEDD4 (100) and PARPs (46) have been proposed as potential MD interactors. By comparison, only two proteins, SNAPIN and N4BP2L2, have been suggested to interact with the AUD (98), leaving this domain with a sparse interactor landscape and emphasizing the need for future work to define whether additional, infection-relevant host factors are recruited by the AUD.

## MOSQUITO HOST FACTORS THAT INTERACT WITH nsP3

Although infection of the mosquito host is essential for alphavirus transmission to vertebrates, few studies have examined nsP3 interactions with mosquito proteins (Table 3) (5, 25, 26, 28, 68, 122).

**TABLE 3:** Mosquito alphavirus nsP3 interactors

Mosquito protein interacting with nsP3	Virus	Study type	System	Reference(s)
Rasputin/RIN	SINV	Affinity purification/co-IP; mass spectrometry	C7/10	(68)
	CHIKV	Co-localization; mutational mapping; functional knockdown	U4.4; adult *Aedes albopictus* mosquitoes	(26)
	CHIKV	AP-MS screen; targeted IP/Western validation; functional genetics	C7/10	(28)
	CHIKV, MAYV, ONNV, SFV, SINV, RRV, SESV	Co-localization microscopy panel	C6/36	(5)
	CHIKV, ONNV	Comparative IP-MS screen	Sua 4.0	(25)
HSC70	SINV	Affinity purification/co-IP; mass spectrometry	C7/10	(68)
BIN 1 (A0A182G3T6)	CHIKV	AP-MS screen; targeted IP/Western validation; functional genetics	C7/10, Aag2	(28, 122)
RM62F	CHIKV	Pull-down/MS screen; co-IP validation	Aag2	(122)
WDR48	ONNV	Comparative IP-MS screen	Sua 4.0	(25)
HIP1	ONNV	Comparative IP-MS screen	Sua 4.0	(25)
Cathepsin L	ONNV	Comparative IP-MS screen	Sua 4.0	(25)
Fermitin	ONNV	Comparative IP-MS screen	Sua 4.0	(25)
BRMS1L	ONNV	Comparative IP-MS screen	Sua 4.0	(25)
28S ribosomal protein S22	ONNV	Comparative IP-MS screen	Sua 4.0	(25)
PABP	CHIKV	Comparative IP-MS screen	Sua 4.0	(25)
COPB1	CHIKV	Comparative IP-MS screen	Sua 4.0	(25)
COPB2	CHIKV	Comparative IP-MS screen	Sua 4.0	(25)
PDHB	CHIKV	Comparative IP-MS screen	Sua 4.0	(25)
40S ribosomal protein S23	CHIKV	Comparative IP-MS screen	Sua 4.0	(25)
40S ribosomal protein S26	CHIKV	Comparative IP-MS screen	Sua 4.0	(25)
Cysteine tRNA ligase	CHIKV	Comparative IP-MS screen	Sua 4.0	(25)
Replication factor C subunit 3/5	CHIKV	Comparative IP-MS screen	Sua 4.0	(25)
Replication factor C subunit 2/4	CHIKV	Comparative IP-MS screen	Sua 4.0	(25)
α-1,4 Glucan phosphorylase	CHIKV	Comparative IP-MS screen	Sua 4.0	(25)
Actin	CHIKV, ONNV	Comparative IP-MS screen	Sua 4.0	(25)
Calcium-transporting ATPase, sarcoplasmic/endoplasmic reticulum type	CHIKV, ONNV	Comparative IP-MS screen	Sua 4.0	(25)
GTP-binding nuclear protein	CHIKV, ONNV	Comparative IP-MS screen	Sua 4.0	(25)
CDK1	CHIKV, ONNV	Comparative IP-MS screen	Sua 4.0	(25)
60S ribosomal protein L28	CHIKV, ONNV	Comparative IP-MS screen	Sua 4.0	(25)
60S ribosomal protein L8	CHIKV, ONNV	Comparative IP-MS screen	Sua 4.0	(25)
U6 snRNA phosphodiesterase	CHIKV, ONNV	Comparative IP-MS screen	Sua 4.0	(25)
Lethal (2) essential for life protein	CHIKV	Comparative IP-MS screen	Aag2	(122)
Trim33	CHIKV	Comparative IP-MS screen	Aag2	(122)
Eiger	CHIKV	Comparative IP-MS screen	Aag2	(122)
Pumilio	CHIKV	Comparative IP-MS screen	Aag2	(122)
Soma ferritin	CHIKV	Comparative IP-MS screen	Aag2	(122)
Rab2	CHIKV	Comparative IP-MS screen	Aag2	(122)
Septin 2	CHIKV	Comparative IP-MS screen	Aag2	(122)
Cytochrome C oxidase 6A	CHIKV	Comparative IP-MS screen	Aag2	(122)
Protein fork head	CHIKV	Comparative IP-MS screen	Aag2	(122)
Crk	CHIKV	Comparative IP-MS screen	Aag2	(122)
Ribosomal protein L6	CHIKV	Comparative IP-MS screen	Aag2	(122)
ATP dependent RNA helicase DBP2	CHIKV	Comparative IP-MS screen	Aag2	(122)
Larval cuticle protein	CHIKV	Comparative IP-MS screen	Aag2	(122)
Ribosomal protein L14	CHIKV	Comparative IP-MS screen	Aag2	(122)
ATP synthase blw	CHIKV	Comparative IP-MS screen	Aag2	(122)
SCAR	CHIKV	Comparative IP-MS screen	Aag2	(122)
Delta4-sphingolipid-FADS-like protein ifc	CHIKV	Comparative IP-MS screen	Aag2	(122)
Tapdelta	CHIKV	Comparative IP-MS screen	Aag2	(122)
E3 ubiquitin-protein ligase ctrip	CHIKV	Comparative IP-MS screen	Aag2	(122)

Validated nsP3 interactions with mosquito host factors are mediated by its HVD (24, 28).

Among the best-characterized mosquito host factors is rasputin (RIN), the mosquito ortholog of G3BP1/2, which binds directly to the nsP3 HVD (5, 25, 26, 28, 68). Similar to vertebrate G3BP1/2, specific FGDF amino acid motifs within the HVD engage the NTF2L domain of RIN (26, 28, 71). For CHIKV, a single FGDF motif is sufficient for infection and dissemination within the mosquito, whereas in the vertebrate host, two FGDF motifs are required for efficient transmission (24). In mosquito infection models, the RIN-FGDF interaction helps alphaviruses evade antiviral responses in the mosquito midgut, and RIN silencing significantly reduces virus infection rates and transmission potential in *Aedes* mosquitoes (26). However, the role of RIN in mosquito cells is less straightforward. Mutagenesis studies suggest that the viral RIN-binding FGDF motif is important for CHIKV replication in C6/36 and C7/10 mosquito cells (28, 123), whereas RIN knockdown in U4.4 cells did not show a replication defect. Residual RIN levels after knockdown may be sufficient for replication, or other mosquito proteins might compensate by interacting with the FGDF motif. Although the mechanisms of RIN in alphavirus infection are unclear, its similarity to vertebrate G3BPs suggests that it may support early steps of infection, including viral RNA binding, recruitment of the 40S ribosomal subunit, vRNA translation, and replication-complex assembly (26, 30, 52, 65).

Another mosquito protein, BIN1, interacts directly with nsP3 via proline-rich regions of the HVD. In mosquito cells, the strongest evidence comes from a CHIKV study showing interaction between nsP3 HVD and the mosquito BIN1 homolog in C7/10 cells and reduced replication after mutation of the BIN1-binding motif (28). However, the role of BIN1 may be context-dependent, since the same motif may also recruit other SH3-domain proteins (5), and anopheline BIN1 was not detected among nsP3 interactors in *Anopheles* Sua 4.0 cells (25). The vertebrate BIN1 is recruited by alphavirus nsP3 and supports efficient SFV and SINV RNA replication (53), but it is unknown what roles it plays in supporting viral replication in the mosquito host.

Byers and colleagues screened anopheline Sua 4.0 cells for host proteins interacting with CHIKV and ONNV nsP3 via transient expression, immunoprecipitation, and mass spectrometry, reproducibly identifying 24 proteins, including both virus-specific and shared interactors (25) (Table 3). Kumar and colleagues screened using purified CHIKV nsP3 incubated in uninfected *Aedes aegypti* cell lysate, followed by immunoprecipitation and mass spectrometry, and identified interactors with a wide range of functions, including roles in cellular translation, ubiquitination, RNAi, and cell signaling pathways (122). Many of the identified mosquito nsP3 interactors in these screens are completely uncharacterized and have no known function.

## nsP3 AS A DETERMINANT OF HOST RANGE SPECIFICITY

nsP3 interactors in vertebrates and mosquitoes shape viral host range specificity. Most alphaviruses are transmitted in cycles between vertebrate and arthropod hosts and require mechanisms that allow the virus to establish productive infection in these disparate hosts. A key mechanism by which nsP3 influences host range is the regulation of viral protein production through a stop codon in its HVD, which regulates full-length nsP1234 production via type II ribosomal readthrough (124, 125). In most gRNA molecules, translation terminates at the stop codon, resulting in the production of the nsP123 polyprotein. However, ribosomes can read through this stop codon at a rate of 10%–30% by incorporating either tryptophan or arginine instead of terminating translation (126), resulting in the production of the complete nsP1234 polyprotein, which is essential for virus replication. The genomes of most alphaviruses contain this stop codon, though naturally occurring alphaviruses lacking this feature also exist: most species of aquatic alphaviruses, most strains of ONNV and SFV, and some strains of Getah virus, RRV, and CHIKV (127).

In SINV, replacing the opal stop codon with a sense codon causes an excess of the full-length P1234 polyprotein, overwhelms nsP2 protease functions, and delays positive-sense vRNA synthesis (127). At 37°C, the presence of the wild-type opal stop codon makes virus replication more efficient in Vero cells, as substitution with a sense codon results in aberrant polyprotein processing (127). This effect is less pronounced at lower temperatures (27°C), and in *Ae. albopictus* mosquito C6/36 cells (127). Another study showed that opal stop codon mutation delays nsPs processing, disrupts replication spherules, and increases Dicer-2 cleavage of SINV RNA, enhancing the antiviral siRNA response (128).

Studies of different alphaviruses have revealed how this stop codon affects viral fitness. *In vivo* studies of ONNV indicate that the opal stop codon promotes virus fitness in its natural *Anopheles* mosquito vectors (129). ONNV with the opal stop codon has been observed to infect twice as many mosquitoes at the same oral dose as viruses containing arginine. In this system, it was also observed that the virus disseminates earlier from the mosquito midgut (129). However, when ONNV was repeatedly passaged in vertebrate Vero cells, the opal codon at the nsP3–nsP4 junction was replaced by an arginine codon (CGA) (130), indicating that the selective pressures in vertebrate cells favor the sense codon. It remains unclear whether the contrasting results between using SINV or ONNV stem from inherent differences among alphaviruses or from other experimental conditions.

Together, these studies indicate that the evolutionary role of the opal stop codon may vary between different alphaviruses and hosts. SINV with the opal stop codon has better fitness at higher temperatures, whereas ONNV requires it primarily for efficient replication in mosquitoes (127, 129). These results highlight potentially divergent roles of the opal stop codon across alphavirus species, which may reflect different evolutionary paths of opal stop codon in different alphaviruses.

Another key mechanism by which nsP3 alters host range specificity is through its interactions. The nsP3 from 11 different alphaviruses was screened for colocalization with human G3BP1 and its mosquito ortholog, RIN, using transient expression of eGFP-tagged nsP3 constructs (5). This experiment revealed that in almost all dual-host alphaviruses, the nsP3 strongly colocalizes with both G3BP1 and RIN (5). In alphaviruses with vertebrate and arthropod transmission cycles, nsP3 was observed to colocalize with RIN under transient plasmid expression, including Southern Elephant Seal Virus, which is believed to be transmitted by aquatic lice rather than mosquitoes (131). Except for nsP3 of VEEV and Tai Forest alphavirus (TALV), all other nsP3s tested colocalized with G3BP1 (5). TALV infects only mosquitoes and has no known vertebrate host and showed nsP3 localization with RIN. Another exception is the nsP3 of Salmonid alphavirus, a salmon-infecting virus, which failed to colocalize with both G3BP1 and RIN (5), contrasting with all other alphaviruses tested. Beyond G3BP1, other stress-related proteins are found in this interaction network. G3BP1 facilitates the colocalization of nsP3 with the FMR1, except in the case of nsP3 of encephalitic alphaviruses, which can bind directly to FMR1, a member of the FXR protein family (52). These studies demonstrate that the ability of nsP3 to colocalize with SG proteins is broadly conserved among alphaviruses that circulate between vertebrate and insect hosts (5, 52).

**Fig 4:**
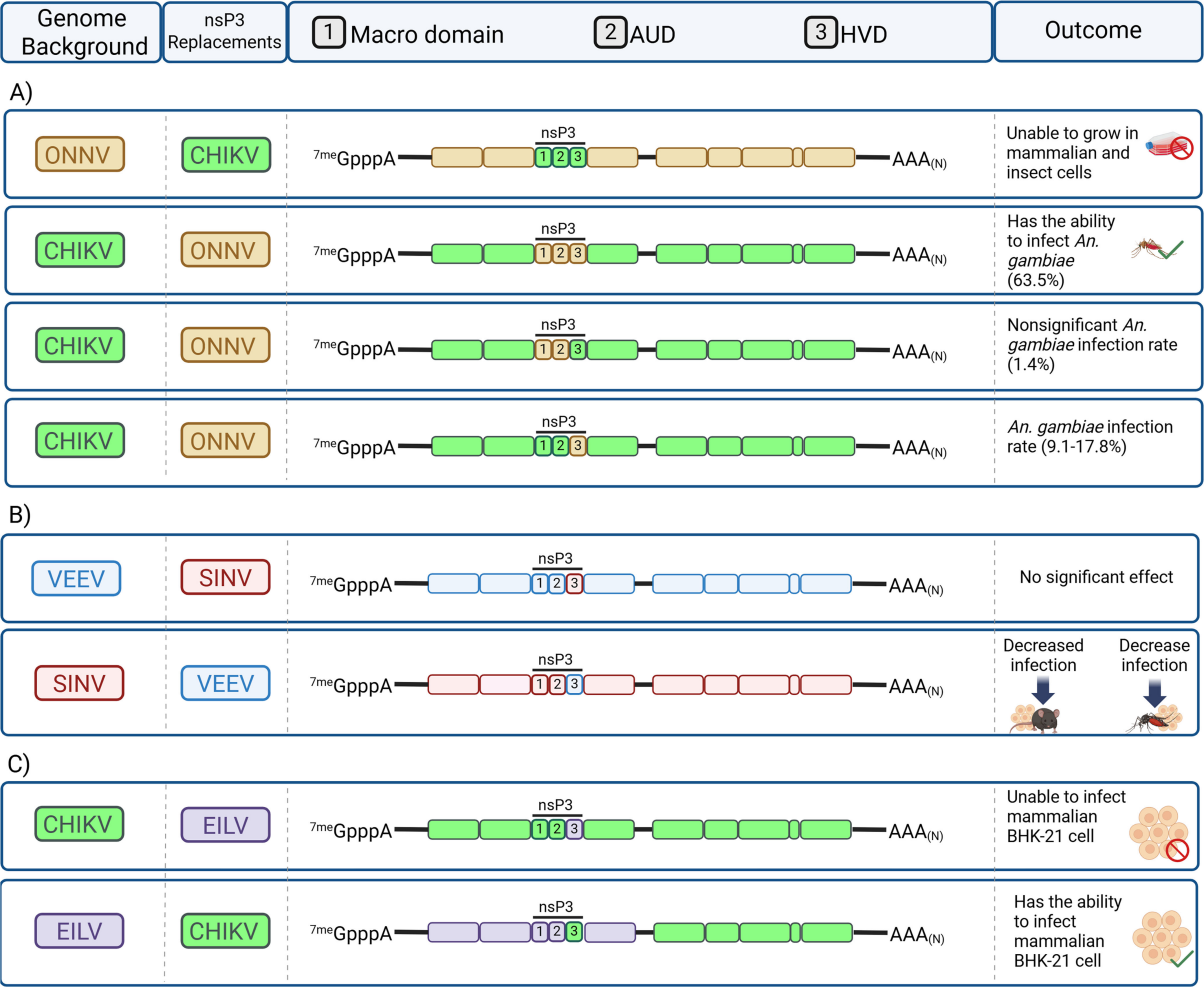
Chimeric virus experiments reveal nsP3 as a critical determinant of the specificity of infection in mosquito versus vertebrate hosts. (**A**) In contrast with CHIKV and several other alphaviruses, which are transmitted by mosquitoes of the *Aedes* genus, ONNV is transmitted by mosquitoes of the *Anopheles* genus. A chimeric ONNV containing CHIKV nsP3 cannot infect mammalian and mosquito cells. In contrast, a chimeric CHIKV with ONNV nsP3 infects anopheline mosquitoes, but this ability is not present at the same levels when only the HVD of CHIKV is present or when only CHIKV MD and AUD are present. (**B**) Chimeric VEEV that contains SINV HVD had no significant differences in replication when compared to the wild type. A chimeric SINV containing HVD from VEEV exhibits reduced infection in rodent and mosquito cells. (**C**) EILV is a mosquito-specific alphavirus. A chimeric CHIKV with HVD from EILV cannot infect vertebrate cells. Chimeras containing the structural proteins of CHIKV and nonstructural proteins of EILV with HVD from CHIKV can infect vertebrate BHK-21 cells.

**Fig 5:**
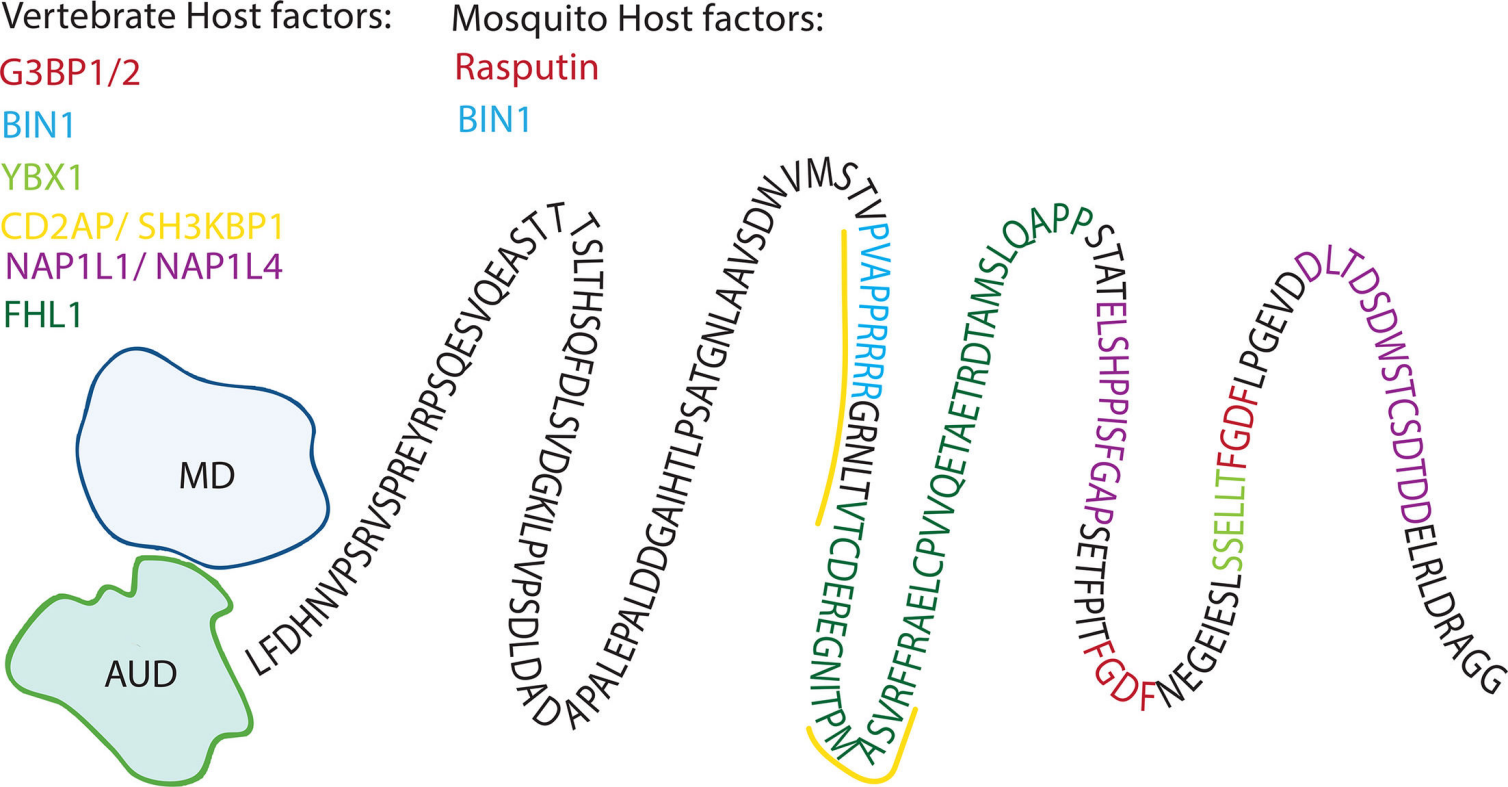
Known nsP3 HVD protein-binding motifs in CHIKV. The HVD contains multiple motifs that bind vertebrate and mosquito proteins, with characterized motifs mainly interacting with pro-viral factors. Protein names from vertebrates or mosquitoes are shown in the top left, and their binding motifs are indicated on the amino acid sequence using matching colors or with colored underscores when motifs overlap. G3BP1/2 and RIN are depicted with the same color because they are orthologs.

Several studies have generated chimeric alphaviruses to investigate the effects of different elements and domains of nsP3 on virus replication in various hosts (Fig. 4). This approach has been particularly informative when comparing CHIKV and ONNV, two alphaviruses of great medical importance that differ in vector specificity. While ONNV is the only alphavirus that is transmitted by *Anopheles* mosquitoes to humans (132), CHIKV is primarily transmitted to humans by *Aedes* mosquitoes, which are known for their ability to transmit a wide range of pathogens. Although *Anopheles* mosquitoes are not competent vectors of CHIKV (133), a chimeric CHIKV carrying ONNV nsP3 can infect adult *Anopheles gambiae* mosquitoes (Fig. 4A), demonstrating that nsP3 is a critical determinant of infection efficiency in mosquitoes (132). In contrast, chimeric ONNV containing the CHIKV nsP3 is unable to replicate in vertebrate or mosquito cell culture (132). The role of HVD in virus replication was also tested using SINV and VEEV constructs (Fig. 4B) in reciprocal nsP3 HVD swap experiments (67). Both chimeras remained viable and replication competent. VEEV carrying SINV HVD replicated similarly compared to wild-type virus in BHK-21 and C7/10 cells and at a slightly higher rate in NIH T3 cells, while SINV carrying the VEEV HVD showed reduced replication efficiency across all the cell lines tested (67). Chimeric viruses were also generated using Eilat virus (EILV) and CHIKV (Fig. 4C). EILV is an insect-specific alphavirus that cannot infect or be transmitted to any vertebrate host or replicate in vertebrate cell lines (134), whereas CHIKV can infect both mosquitoes and vertebrates. A chimeric CHIKV containing the HVD of nsP3 from EILV can infect mosquito cells but cannot infect hamster BHK-21 cells (28). In contrast, chimeric EILV containing CHIKV structural proteins and the HVD of nsP3 can infect both mosquito and BHK-21 cells. These findings suggest that nsP3, and especially the HVD, can serve as an important determinant of host specificity (28). Certain regions of the nsP3 HVD are essential for CHIKV replication in both hamster and mosquito cells, whereas other regions in the HVD differentially affect virus replication in mosquito and vertebrate cells (Fig. 5) (28). This host-specific response suggests that vertebrate and mosquito factors likely interact with distinct regions of the HVD, indicating different binding priorities for each species (28).

## DISCUSSION AND FUTURE RESEARCH DIRECTIONS

Despite advances in our understanding of the alphavirus nsP3 structure, subcellular localization, interactors, and its role in the virus replication cycle, many gaps in knowledge remain. A vastly underexplored field involves the study of nsP3-mosquito interactions. Genome annotation and reagents for mosquitoes are not well standardized, with varying quality across mosquito species, such that even when a homolog of an important nsP3 interactor is identified, its functional conservation is unverified. Another limitation is that the existing efforts to identify mosquito interactors of nsP3 use cell lines, most of which are derived from larval stages, and do not specifically model any mosquito organ. Many of these are also incompetent for RNAi, the main branch of the mosquito antiviral response. These cells may not reflect natural virus-mosquito interactions. For example, RIN (mosquito G3BP1 ortholog) binds CHIKV nsP3 and promotes infection in *Ae. albopictus* mosquitoes, but is dispensable in U4.4 cells (26). Understanding nsP3 roles in mosquito infection will require a better model system, improved genome annotation of important vector species, and more *in vivo* screens and mechanistic studies to analyze nsP3 function.

Another critical gap in alphavirus nsP3 biology is the scarcity of mechanistic studies of its domains and respective interactors. There is a critical need to identify the ADPr cellular targets of MD for binding and removal of these modifications, and the mechanisms by which these functions affect virus replication. It is also a priority to investigate the binding sequence, structure, or other specificity of the AUD-RNA interactions, and to derive a high-resolution structure of AUD in complex with vRNA to understand how these interactions contribute to vRNA replication. HVD’s structural dynamics upon partner binding also require further investigation, alongside systematic motif mapping, to reveal how nsP3 engages host factors and possible methods for disrupting these interactions. Integrating structural studies of HVD in complex with host interactors and targeted mutagenesis studies will provide a deeper understanding of HVD interaction dynamics.

Further research is also needed to deepen understanding of the roles of SG and VMG during alphavirus infections, as well as the mechanisms by which nsP3 modulates them. To help clarify nsP3’s contribution, additional research should focus on the mechanistic and temporal roles of nsP3 in the transition from early SG to VMG. Based on the available data, this transition is likely initially driven by alphavirus-induced transcriptional and translational shutoffs (62). In addition, MD also mediates removal of ADP-ribose from G3BP1 (45), and HVD prevents G3BP1 from participating in canonical SG (52, 60, 71, 72). Viral capsid accumulation may also assist in maintaining this transition, but the concentration of capsid required indicates that it would function only at later stages of infection. These mechanisms indicate temporal regulation of the transition from SG to VMG, and the redundancy of these mechanisms underscores the potential importance of this process for virus replication.

nsP3 is highly multifunctional, and its contributions to viral emergence remain incompletely defined. Studies sequencing and testing outbreak viruses have found that the region coding for nsP3 is a common site of variation (130, 135–143), and in some instances, phenotypic differences from earlier circulating strains have been validated (135, 144–146). In most cases, the biological significance and contribution of specific nsP3 changes to emergence remain unresolved, and the causal link between these mutations and altered outbreak risk has not been established. Laboratory and field studies are both needed to elucidate differences in host specificity, viral fitness, and other mechanisms governing epidemic potential among these naturally occurring nsP3 variants in natural settings.

In summary, increased understanding of the functions, structure, and interactome of nsP3 and its domains is essential for elucidating the molecular strategies alphaviruses use in their evolutionary arms race to replicate in both mosquito and vertebrate hosts. Further studies in these areas have great potential to unlock new fundamental insights into virus and cell biology, hence enabling the development of tools to improve the prediction of viral emergence, guide the development of treatment strategies, and target and block transmission by mosquito vectors.
